# Supplementary material for: Dutasteride treatment and its effect on standardized uptake values in prostate-specific membrane antigen-PET imaging: A pilot study
Source: PLoS One. 2026 May 6;21(5):e0348695. doi: 10.1371/journal.pone.0348695 (PMC13148650; doi:10.1371/journal.pone.0348695)
Supplement: Attachment — Submitted filename: Response to Reviewers PLOS 18.03.26.docx [file pone.0348695.s002.docx]

**Rebuttal letter**

**Response to reviewer 1:**

Despite the small cohort, this is an interesting and well-written study that assesses the impact of short-term Dutasteride on PSMA expression based on PSMA PET/MRI. Here are my comments:

“This type II transmembrane glycoprotein is overexpressed in PCa cells”. Precisely in around 90% due to intrinsic PCa heterogeneity, see PMID 40615466 and 39083067

- Thank you for your feedback and this helpful comment. We have revised the manuscript accordingly and incorporated this clarification into the relevant section.

“Even though modern PSMA PET/CTs have a superior diagnostic accuracy than conventional imaging (4), the detection rate remains limited in patients with low-volume disease (low-grade cancer, low PSA) at the time of recurrence (5-7).
Conversely, salvage radiotherapy is most effective in patients with PSA levels below 0.2 ng/mL (8-10).”

Indeed, as per the EAU guideline, a negative PSMA PET should not delay salvage RT.

- Thank you for this valuable comment. The manuscript has been revised accordingly.

“Due to the potential side effects (13) of these compounds, recent studies have investigated the impact of dutasteride on PSMA expression in vitro and in vivo (14, 15)”. We now have initial data already reported in an expert paper, such as Vaz S et al. Influence of androgen deprivation therapy on PSMA expression and PSMA-ligand PET imaging of prostate cancer patients. Eur J Nucl Med Mol Imaging. 2020;47:9–15. 10.1007/s00259-019-04529-8.

This can be used to extend the introduction (i.e., discussing the hormone therapy's different impact on HSPC and CRPC), also dealing with potential early use of radioligand therapy in the discussion section (i.e., PMID 39934300)

- Thank you for this great suggestion. We have incorporated the relevant information into the manuscript. Regarding the impact of ADT and AR pathway inhibitors on HSPC and CRPC, we respectfully chose not to expand on this aspect, as it is not directly related to the focus of the present study and would extend beyond the scope of the manuscript. The early use of radioligand therapy is indeed a very interesting perspective; however, further studies are required to better evaluate this therapeutic approach.

Please describe which Specialists are involved in the interdisciplinary tumour board at the University Hospital of Zurich.

- Thank you for this comment. We added this important information to the manuscript.

In the whole paper, use EANM radiopharmaceuticals nomenclature (i.e. 68Ga-PSMA-11 à [68Ga]Ga-PSMA-11)

- Thank you for this clarification. We have revised the manuscript to use the correct nomenclature.

How did the authors select the administered radiopharmaceutical dose?

- Great question. Based on previous work (Ferraro DA et al 2021, EJNMMI 2021, PMID: **33620559)** with PSMA PET/MRI of the pelvis for biopsy guidance we had experience with low dose PET. Given the long acquisition time for the pelvic MRI the dose of 75-89 MBq PSMA is sufficient for excellent image quality.

In the whole paper, use only one term among “MRI” or “MR”.

- Great input. We revised the manuscript in that regard.

Add the initials and years of experience of the dual board-certified radiologist and nuclear medicine
Physician, the specialised uropathologist, as well as the second board-certified pathologist.

- Thank you for this valuable comment. We have incorporated this information into the manuscript.

Did the authors also consider assessing SUVmean and volumetric PSMA PET parameters?

- Great comment. Yes, we did observe the same trend; however, the values were somewhat less robust (R1/R2). Therefore, we decided to focus on SUVmax, which is more widely accepted and more strongly associated with tumor aggressiveness and PSMA expression.

For your information:

| **Patient** | **Lesion** | **R1 SUVmax_1 / Mean_** | **R2 SUVmax_1 / mean_** | **R1 SUVmax_2 / Mean_** | **R2 SUVmax_2 / Mean_** |
| --- | --- | --- | --- | --- | --- |
| **1** | 1 | 3.1 / 2.0 | 2.9 / 2.2 | 3.3 / 2.4 | 3.3 / 2.6 |
| 2 | 2 | 7.2 / 4.1 | 7.2 / 4.5 | 9.7 / 5.6 | 9.7 / 5.8 |
|  | 3 | 8.0 / 4.7 | 8.0 / 4.5 | 7.1 / 4.1 | 7.2 / 4.2 |
| 3 | 4 | 7.5 / 4.2 | 7.5 / 4.4 | 8.3 / 4.8 | 8.3 / 4.0 |
|  | 5 | 7.0 / 5.0 | 7.0 / 5.0 | 7.3 / 5.2 | 7.3 / 5.3 |
| 4 | 6 | 5.5 / 3.5 | 5.5 / 3.5 | 5.7 / 3.6 | 5.7 / 3.6 |

Describe in a little more detail the statistical section: did the authors assess the distribution of each parameter? 68.8 years (66-72) à i.e., it seems that you used the mean and the range, which is not proper.

- Thank you again for your helpful feedback. We have revised and improved the statistical section accordingly.

PI RADS 3 is not suspicious but uncertain.

- Thank you for pointing this out. We have corrected the term to ensure greater precision.

Were there any PSMA PET-negative PCa lesions?

- Great question. All cancerous lesions are displayed in Figure 2. There was one biopsy core showing ISUP grade 1 in the first patient. This biopsy core is located anterior to the PSMA PET lesion.

Another point of discussion may be the different % increase between PCa lesion and background before and after dutasteride.

- Thank you again for your valuable feedback. We have revised the manuscript accordingly, which has helped to improve its overall quality.

Did the authors use a standard VOI? Or was it adapted based on each lesion's dimensions?

- Great question. The VOI size was adjusted according to the dimensions of each lesion. We incorporated this information into the manuscript.

In patients 2 and 3 with two lesions, no malignant tissue was found in the second PSMA-positive lesions 3 and 5.” Potentially misleading, please rephrase.

- Thank you for this helpful comment. We agree that the sentence could be misleading and have therefore revised the wording in the manuscript to improve clarity.

The first lines of the discussion should be reorganised in a more proper form as they are more similar to the results description.

- Thank you for this helpful comment. We agree and have revised the first lines of the Discussion accordingly.

“Despite this discrepancy, dutasteride treatment increased SUVmax in this lesion” I would add, “with less pronounced effect on the normal prostatic tissue”.

- We have added this clarification to the manuscript.

**Response to reviewer 2:**

Dear Authors,
your manuscript is very interesting and shows excellent potential. The paper is well written and reads smoothly; the methods are clearly described and detailed. Although the results are based on only four patients, they are appropriately discussed.

However, I would like to raise a few points.

Why did you not consider using, as background, a tissue that is likely less sensitive to the drug and more suitable as a reference standard, such as the salivary glands, rather than the contralateral side of the prostate gland?

- Thank you for this valuable suggestion. In principle, the use of a reference tissue that is less likely to be influenced by the investigated drug, such as the salivary glands, could indeed provide a more stable internal reference. However, in our study the PSMA PET/MR acquisition protocol was limited to the prostate region and did not include whole-body imaging. This approach was chosen for ethical and financial reasons, as the study population consisted primarily of patients with favorable intermediate-risk prostate cancer, for whom additional whole-body imaging was not clinically indicated. Only one patient had an unfavorable intermediate-risk prostate cancer. Consequently, organs such as the salivary glands were not available for quantitative comparison. For this reason, we used the contralateral side of the prostate as an internal reference within the available imaging field. We acknowledge that alternative reference tissues might provide additional insights, and future studies including whole-body PSMA PET imaging could help further clarify the systemic effects of dutasteride on PSMA-ligand uptake in different organs.

In general, I believe that the limitations of the results should be highlighted more clearly.

- Thank you for this helpful comment. We have revised the Discussion section to more clearly highlight the limitations of our results.

**Response to reviewer 3:**

The pilot study by Praetzel et al. explores the effect of dutasteride on PSMA PET. Despite the small sample size, which limits the ability to draw definitive conclusions, as the authors clearly state, I find the manuscript to be well structured and methodologically sound. It is an excellent starting point for future studies, given the scientific rationale underlying the article and its potentially significant clinical utility.

- Thank you very much for your thoughtful feedback and for the time you devoted to reviewing our manuscript.
